# Editorial: New Insights In Anaphylaxis

**DOI:** 10.3389/fimmu.2018.00506

**Published:** 2018-03-14

**Authors:** Carlos Pastor-Vargas, Vanesa Esteban

**Affiliations:** ^1^Department of Immunology, Instituto de Investigación Sanitaria Hospital Universitario Fundacion Jimenez Diaz (IIS-FJD, UAM), Madrid, Spain

**Keywords:** anaphylaxis, drug and food allergy, molecular signaling, augmentine factors, endothelial cells

**Editorial on the Research Topic**

**New Insights In Anaphylaxis**

In choosing a theme for this issue of *Frontiers in Immunology*, we settled on anaphylaxis, which is understood as the most aggressive manifestation of allergic disorders, and also one that has often been studied solely from an immunological point of view. Recent insights, however, suggest that different perspectives are necessary to advance the knowledge of this multifactorial and complex clinical condition. The articles published here offer a wide-angle view of anaphylaxis, from diagnosis and treatment, through the augmenting factors and cofactors involved, and including the study of the molecular and cellular mechanisms underlying anaphylactic reactions.

Drugs are the most common cause of anaphylaxis in adults. Here, Montanez et al. present an extensive knowledge update on this issue, providing in-depth analysis of the manifold aspects of the two most common anaphylaxis-causing drugs, i.e., non-steroidal anti-inflammatory drugs (NSAIDs) and antibiotics. The study, which includes the latest epidemiological data, molecular mechanisms, and risk factors, offers a brief guide on drug-allergy diagnosis using both *in vivo* and *in vitro* testing methods. On a related topic, a chapter by Castells details new insights into drug allergy and anaphylaxis in the context of cancer and chronic inflammatory diseases. In this work, current knowledge in personalized desensitization protocols is detailed and aimed at the management of patients who have suffered reactions to their first-line therapy. In this context, personalized protocols are a promising first step to enhance the quality of life and life expectancy of patients.

Food allergy is a serious health concern and its prevalence is on the rise, especially in the pediatric population. Allergy to food products is a frequent cause of anaphylaxis and one that is significantly detrimental to patient quality of life. This paper presents the most relevant aspects of food allergy and anaphylaxis. In this contribution, we see the complex network and multiple factors that can affect food-induced anaphylaxis such as genetic predisposition, environmental factors, and the influence of dietary or intestinal micro biota (Benede et al.). Moreover, key features of the immunological mechanisms of the intestinal mucosa involved in food allergy and food-induced anaphylaxis are illustrated. As the authors remark, despite the efforts exerted in this area of research, much more investigation is needed to mitigate this disease.

In human anaphylaxis, the available information on the cellular and molecular level mainly points to the immunological processes that represent the early stages of the anaphylactic reaction. Overall, these events are triggered by hypersensitivity reactions mediated by FcεR1-bound IgE antibodies in response to allergens and result in activation of mast cells (MCs) and basophils. These immune cells are considered the main cell effectors and amplifiers of the allergic reaction. MCs activation induces the release of mediators, which are the ultimate elicitors of reactions in the resident tissues of the surrounding area. As anaphylaxis in patients with systemic mastocytosis is up to 100 times more frequent in patients with systemic mastocytosis, an overview of the epidemiology, triggers, and risk factors of anaphylaxis in patients with MC activation syndromes is included (Gonzalez-de-Olano and Alvarez-Twose). Moreover, the diagnosis and treatment aspects related to this syndrome are given in-depth treatment.

The identification of other cell types participating in anaphylaxis, such as neutrophils or macrophages, is especially relevant to experimental anaphylaxis. Mice deficient in either MCs or IgE still develop anaphylaxis, thereby suggesting the existence of alternative pathways (to date, mainly IgG). Focusing on this molecular and cellular aspect, Escribese et al. report on the potential role of macrophages in anaphylaxis and how these can play an important role in IgG-dependent anaphylaxis induced by allergen-IgG immunocomplex bound to IgG receptors on macrophages, neutrophils, and basophils. Knowledge of other molecular mechanisms that impact the anaphylactic process enables us to speculate as to whether the known classical effector cells are the only meaningful contributors also to the human anaphylactic episodes.

Recent findings on cofactors and augmenting factors are of great importance when establishing degrees of risk in anaphylaxis patients, as these factors may predict or even prevent the occurrence of reactions. Some such factors include estrogens, exercise, statins, alcohol, drugs such as NSAIDs, angiotensin-converting enzyme inhibitors, and β-blockers (Munoz-Cano et al.). This collection contains a number of descriptions detailing the most common immunological mechanisms occurring in anaphylaxis, though special emphasis is given to non-immune mechanisms. One example is the interesting review by Poulsen et al., whose chapter focuses on the importance of internal dose, described as the quantity of the anaphylactic trigger (most often an allergen), allergokinetics as a mechanistic factor, and also the importance of intrinsic and extrinsic cofactors in the pathophysiology and occurrence of anaphylactic reactions.

New findings on the potential of molecular mechanisms are essential to better understand diverse inflammatory-related pathologies linked to allergy and anaphylaxis. This collection of articles includes two studies providing support for the relevance of the contact, complement, and coagulation systems (Bender et al.; Guilarte et al.). Activation of the FXII substrate triggers the kallikrein system, releases the mediator bradykinin, and activates the complement pathway. The relevance of FXII in hereditary angioedema and anaphylaxis highlights this molecule and its related pathways as targets in current diagnosis and treatment. In addition, anaphylaxis mediators are also involved in the activation of the fibrinolytic and coagulation system. Therefore, although only histamine and tryptase can be routinely measured as biomarkers in clinical practice, use of reliable biomarkers to assess the activation of these systems together with standardized assays would ameliorate the diagnosis and management of this disease.

Finally, these relevant pathways connect MCs activation with the mediator’s ability to increase inflammation and permeability/contractility processes occurring in vessels. Regarding vessels and anaphylaxis, original data supported from our own studies reveal Rcan1 an endothelial protein synthesized in response to histamine, which contributes to the strengthening of the endothelium in response to anaphylaxis (Ballesteros-Martinez et al.). This new insight underscores the potential of endothelial molecules as regulators of sensitivity to anaphylaxis.

We are thankful to the medical doctors, researchers, and colleagues who have contributed to this issue, as it would not have been possible without their highly valuable expertise in allergy and anaphylaxis research.

## Author Contributions

CP-V and VE wrote and edited the manuscript.

## Conflict of Interest Statement

The authors declare that the research was conducted in the absence of any commercial or financial relationships that could be construed as a potential conflict of interest.

